# Characterization of a novel species-specific 51-amino acid peptide, PEP51, as a caspase-3/7 activator in ovarian follicles of the ascidian, *Ciona intestinalis* Type A

**DOI:** 10.3389/fendo.2023.1260600

**Published:** 2023-09-29

**Authors:** Tsubasa Sakai, Tatsuya Yamamoto, Takehiro Watanabe, Akiko Hozumi, Akira Shiraishi, Tomohiro Osugi, Shin Matsubara, Tsuyoshi Kawada, Yasunori Sasakura, Toshio Takahashi, Honoo Satake

**Affiliations:** ^1^ Bioorganic Research Institute, Suntory Foundation for Life Sciences, Kyoto, Japan; ^2^ Shimoda Marine Research Center, University of Tsukuba, Shizuoka, Japan

**Keywords:** test cells, ovarian peptides, apoptosis, ascidian, follicle

## Abstract

Invertebrates lack hypothalamic-pituitary-gonadal axis, and have acquired species-specific regulatory systems for ovarian follicle development. Ascidians are marine invertebrates that are the phylogenetically closest living relatives to vertebrates, and we have thus far substantiated the molecular mechanisms underlying neuropeptidergic follicle development of the cosmopolitan species, *Ciona intestinalis* Type A. However, no ovarian factor has so far been identified in *Ciona*. In the present study, we identified a novel *Ciona*-specific peptide, termed PEP51, in the ovary. Immunohistochemical analysis demonstrated the specific expression of PEP51 in oocyte-associated accessory cells, test cells, of post-vitellogenic (stage III) follicles. Immunoelectron microscopy revealed that PEP51 was localized in the cytosol of test cells in early stage III follicles, which lack secretory granules. These results indicate that PEP51 acts as an intracellular factor within test cells rather than as a secretory peptide. Confocal laser microscopy verified that activation of caspase-3/7, the canonical apoptosis marker, was detected in most PEP51-positive test cells of early stage III. This colocalization of PEP51 and the apoptosis marker was consistent with immunoelectron microscopy observations demonstrating that a few normal (PEP51-negative) test cells reside in the aggregates of PEP51-positive apoptotic test cells of early stage III follicles. Furthermore, transfection of the PEP51 gene into COS-7 cells and HEK293MSR cells resulted in activation of caspase-3/7, providing evidence that PEP51 induces apoptotic signaling. Collectively, these results showed the existence of species-specific ovarian peptide-driven cell metabolism in *Ciona* follicle development. Consistent with the phylogenetic position of *Ciona* as the closest sister group of vertebrates, the present study sheds new light on the molecular and functional diversity of the regulatory systems of follicle development in the Chordata.

## Introduction

Ovarian follicle development is a critical process in animal reproduction. In vertebrates, ovarian follicles consist of a single oocyte and surrounding somatic accessory cells such as granulosa cells and theca cells. Follicle maturation and ovulation processes are regulated by the hypothalamic-pituitary-gonadal axis (HPG axis), which acts on both oocytes and the surrounding accessory cells (1, 2). In contrast, invertebrates lack an HPG-axis including pituitary hormones, gonadotropins, and have evolved unique molecular mechanisms underlying follicle development.

Ascidians are marine invertebrates that belong to the Urochordata in the superphylum, Chordata; they are phylogenetically the closest sister group to vertebrates (3). In the ovarian follicles of the cosmopolitan ascidian, *Ciona intestinalis* Type A (*Ciona robusta*), oocytes are enclosed within layers of outer and inner follicle cells, a non-cellular vitelline coat, and test cells that are dispersed in the egg membrane (4). The development of ovarian follicles of ascidians consists of the following developmental stages: stage I (pre-vitellogenic), stage II (vitellogenic), stage III (post-vitellogenic), and stage IV (mature) (5–7). Our previous studies revealed that a variety of neuropeptides secreted from the cerebral ganglion, such as *Ciona* tachykinin (CiTK), *Ciona* neurotensin-like peptide 6 (CiNTLP6), *Ciona* vasopressin (CiVP), and *Ciona* cholecystokinin (cionin) regulate follicle growth, oocyte maturation, and ovulation in *Ciona* in a developmental stage-specific fashion (8–14). Moreover, the expression of receptors for other neuropeptides in the ovary and/or oocyte-associated accessory cells including test cells (15–17) suggest that other neuropeptides are responsible for the regulation of follicle development.

In vertebrates, oocytes, granulosa cells, and theca cells play pivotal roles in the regulation of follicle growth, maturation and ovulation via secretion of various ovarian factors including growth factors and/or sex steroid hormones via functional interactions with the HPG-axis (18–22). In contrast, ovarian factors involved in regulation of follicle development in *Ciona* have yet to be identified. Previously, we substantiated that CiTK stimulates follicle growth from stage II to stage III via upregulation of gene expression and enzymatic activity of cathepsin D, a protease responsible for processing of vitellogenin, in oocyte-associated accessory cells, test cells that are deemed to be, at least in part, functional counterparts of vertebrate granulosa cells (8, 9). These findings suggest that test cells are involved in follicle development. Nevertheless, the biological role of test cells in follicle growth largely remains to be investigated.

During follicle development in ascidians, test cells, emerging on the surface of oocyte at stage II, are excluded from the surface at late stage III, followed by the formation of a new monolayer of test cells in the perivitelline space between the oocyte and the vitelline coat (23, 24). Notably, *Ciona* test cells exhibit morphological changes, such as the disappearance of secretory granules and the development of large vacuoles between stage II and stage III (23, 25), and of filopodia from the surface after fertilization (25). These findings suggest that test cells exert different functions at each stage of follicle development. In this study, we show the molecular characterization of a novel species-specific 51-amino acid peptide, designated PEP51, localization of PEP51 in test cells, colocalization of PEP51 with an apoptotic marker, caspase-3/7, in test cells at early stage III follicles, and activation of caspase-3/7 in cultured cells by PEP51. Combined with the crucial phylogenetic position of ascidians as the closest relative to vertebrates, the present study suggests that a species-specific ovarian peptide plays key roles in the regulatory mechanisms underlying follicle development in chordates, and is also involved in the evolution and diversification of a follicle development process in an animal.

## Materials and methods

### Animals

Adult *Ciona intestinalis* Type A (*Ciona robusta*) were cultivated in sterile artificial sea water (ASW) at 18°C at the Maizuru Fisheries Research Station at Kyoto University or Misaki Marine Biological Station at the University at Tokyo.

### Antibodies

The 20-amino acid fragment of PEP51 (CSTGNKIYWNKFEQIKSHLY-NH_2_, at amino acid sequence 22–41) was conjugated with keyhole limpet hemocyanin and used as an antigen (Sigma-Aldrich Japan, Tokyo, Japan). Antigen affinity-purified anti-PEP51 antibody raised in rabbits was obtained from Sigma-Genosys (Sigma-Aldrich Japan, Japan). Western blotting analysis confirmed that the anti-PEP51 antibody recognized endogenous PEP51 in the *Ciona* follicles and synthetic PEP51 peptide (Eurofin Genomics, Tokyo, Japan).

### Peptide synthesis

The Full-length sequence of PEP51 peptide (MLSIKSLVSICMVVQSILKNICSTGNKIYWNKFEQIKSHLYNLINQPNKLC) was chemically synthesized by Eurofin Genomics (Tokyo, Japan) with a purity of 99.06%.

### Immunohistochemistry on *Ciona* ovary sections

*Ciona* ovaries were fixed in Bouin’s solution, embedded in paraffin, and cut into 7-μm sections. Preparation and immunostaining of the ovary sections were performed as previously described (15, 16). Immunoreactivity was visualized using the anti-PEP51 antibody (1:1000) and an alkaline phosphatase (AP) conjugated secondary antibody (1:2000) with BCIP-NBT (Nacalai Tesque, Kyoto, Japan) as a chromogen. No specific immunostaining was observed using a secondary antibody alone or the preabsorbed anti-PEP51 antibody. The preabsorbed anti-PEP51 antibody (1:1000) was prepared by incubation with the antigen peptide (CSTGNKIYWNKFEQIKSHLY-NH_2_), which was used to generate the antibody, at a final concentration of 10^-6^ M for overnight at 4 ˚C. All of the immunoreactivity experiments were performed in triplicate.

### Fractionation of *Ciona* follicles

Isolated *Ciona* ovaries were washed with ASW, cut into several pieces, and enzymatically disaggregated with 0.2% (w/v) collagenase in ASW for 15 min at room temperature with orbital shaking. The disaggregated follicles from the ovaries were fractionated using a series of stainless-steel sieves of different mesh sizes (150, 90, 63, 38, and 20 μm; TOKYO SCREEN CO. LTD., Tokyo, Japan) as described previously (6, 7).

### Tricine-SDS-PAGE and western blotting

Proteins in the *Ciona* follicles were extracted using Proteoextract complete proteome extraction kit (Merck Millipore, Burlington, MA, USA) and quantified using the BCA protein assay kit. A 30-μg aliquot of soluble protein or 10-ng synthetic PEP51 peptide were separated by 16.5% tricine-SDS-PAGE (sodium dodecyl sulphate polyacrylamide gel electrophoresis) and transferred to PVDF membranes. The membranes were blocked with 5% skimmed milk in Tris-buffered saline with Tween 20, TBS-T (20mM Tris–HCl, 150mM NaCl, 0.1% Tween 20) at 4°C, and subsequently incubated overnight with rabbit anti-PEP51 antibody (diluted 1:2000) at 4°C. After washing three times with TBS-T, the blots were incubated with an AP-conjugated anti-rabbit IgG secondary antibody (diluted 1:50,000) for 1 h at room temperature. Blots were visualized by BCIP-NBT (Nacalai Tesque) according to the manufacture’s instruction.

### Identification of PEP51 from test cells of stage III follicles

*Ciona* ovaries from five adults were collected and washed with ASW, and the follicles were fractionated as described above. Isolated stage III follicles were enzymatically disaggregated (0.1% trypsin, 0.5% collagenase, and 0.2% actinase E in ASW) for 15 min at room temperature by orbital shaking to obtain single-cell suspensions. After disaggregation, 5% fetal bovine serum (BSA) was added, and cells were passed through a 20-μm cell strainer (Celltrix, Sysmex, Kobe, Japan) and were washed with ASW. Cells were then centrifuged (390 ×g for 2 min at 4°C) and resuspended in a staining solution (1% BSA in ASW) for 5 min. The anti-PEP51 antibody (1:300) and Alexa Fluor 488-conjugated secondary antibody (1:500) in 1% dimethyl sulfoxide (DMSO) containing the staining solution was then added and incubated for 30 min on ice, respectively. After washing with ASW, PEP51-positive (PEP51(+)) cells were sorted using a fluorescence-activated cell sorting (FACS) system (FACSMelody; BD Biosciences, NJ, USA). Subsequently, endogenous peptides in the FACS-sorted cells were extracted in 50% acetonitrile (ACN) containing 0.1% trifluoroacetic acid (TFA) by pipetting. The evaporated extract was treated with reverse phase C8 ZipTip for de-salting (Merck Millipore, Carrigtwohill, Ireland), and the elute was evaporated and concentrated. The peptides on an anchorchip were measured using Bruker rapifleX, matrix-assisted laser desorption/ionization (MALDI) time of flight (TOF) instrument (Bruker Daltonics, Bremen, Germany), and the resultant mass spectrometric data were analyzed using MASCOT database search (Matrix Science, Tokyo, Japan). In addition, *In-gel* tryptic digestion of the band corresponding to a size of 6kDa on tricine-SDS-PAGE of the stage III follicle lysate was analyzed for protein identification using a nanoLC-ESI Q-TOF MS instrument (QSTAR XL, Applied Biosystems, Waltham, MA, USA). The tandem MS (MS/MS) spectrometric data were analyzed using a MASCOT database search (Matrix Science). *In-gel* tryptic digestion and nanoLC-ESI Q-TOF MS/MS analysis was conducted by Japan Proteomics, Inc. (Miyagi, Japan).

### Immunoelectron microscopy

*Ciona* ovaries were fixed in 4% paraformaldehyde (PFA) and 0.1% glutaraldehyde (GA) (Distilled EM grade, Electron Microscopy Sciences, Hatfield, PA, USA) in 0.1M phosphate buffer (PB) pH 7.4 at 4 °C for 1h. After washing, the samples were dehydrated and infiltrated with a 50:50 mixture of ethanol and resin, transferred to a fresh 100% resin (LR white, London Resin, Berkshire, UK), and polymerized by an ultraviolet polymerizer. Ultra-thin sections of the polymerized resins were mounted on nickel grids and incubated with anti-PEP51 antibody, followed by 15nm gold particle-labelled secondary antibody. The grids were placed in 2% GA in 0.1 M PB and dried, then stained with 2% uranyl acetate for 15 min and a Lead stain solution (Sigma-Aldrich, Tokyo, Japan). The samples were observed under a transmission electron microscope (JEM-1400Plus, JEOL, Tokyo, Japan) at 100 kV. Digital images were obtained with a CCD camera (EM-14830RUBY2, JEOL). Immunoelectron microscopy was carried out by Tokai Electron Microscopy, Inc. (Nagoya, Japan).

### Cloning of *Pep51* cDNA from stage III follicles

Total RNA (0.5 μg) extracted from *Ciona* stage III follicles was reverse-transcribed to the template cDNA at 55 °C for 60 min using the gene-specific primer (5’-GGTTCTAACATGTGTGGTCTTG-3’, identical to nucleotides 1009–1030), which was designed according to the sequence of the *Pep51* gene (Ensembl gene ID: ENSCING00000021415) on the Ensembl web browser (http://asia.ensembl.org/Ciona_intestinalis/Gene/). The template cDNA was amplified by PCR using the primers 5’- TTCTACACCCTGCAAACACTGTAAC-3’, identical to nucleotides 310–334, and 5’-CTGATTGTGCTTCGTATCCTG-3’, complementary to nucleotides 988–1008. The PCR product was cloned into the TArget clone vector (Toyobo, Osaka, Japan). Subcloned inserts were sequenced on an Applied Biosystems 3500 Genetic Analyzer (Applied Biosystems, Foster City, CA, USA), using a Big-Dye sequencing kit (Applied Biosystems) and universal primers (T3 and T7 primers).

### PEP51-expression vector construction and transfection

An open reading frame of *Pep51* was amplified using gene-specific primers with extensions to vector ends (Fw; 5’-CCGGAATTCGCCATGTTGAGCATAAAATC-3’, and Rv: 5’- AAAGCGGCCGCTTAGCACAGTTTATTTG-3’, including stop codon), which contain an *Eco*RI site and *Not*I site, respectively. The amplified PCR product and the expression vector pcDNA4/V5 (Thermo Fisher Scientific, Waltham, MA, USA) were digested with *Eco*RI and *Not*I restriction enzymes, followed by ligation using Takara Ligation Mighty Mix (Takara, Shiga, Japan) according to the manufacturer’s instructions. The expression vector, empty pcDNA4 vector (0.1 μg), or transfection medium alone was transiently transfected into 5×10^4^ African green monkey kidney fibroblast cells (COS-7 line) or genetically engineered human embryonic kidney cells (HEK293MSR line) in 9.5-mm wells of multi-well glass-bottom dish (Matsunami, Osaka, Japan) with Lipofectamine 2000 (Thermo Fisher Scientific), according to the manufacturer’s instructions. COS-7 cells and HEK293MSR cells were grown under 5% CO_2_ at 37°C in Dulbecco’s modified Eagle’s medium (DMEM) supplemented with 10% heat-inactivated FBS, and DMEM supplemented with 10% heat-inactivated FBS and 1% non-essential amino acids, respectively. Transfection efficiency (approximately 60-70%) was evaluated by an immunocytochemistry as described below.

### Detection of apoptosis in PEP51-transfected cells

After transfection of the *Pep51* expression vector into COS-7 cells and HEK293MSR cells for 2 days at 37°C, a cell-permeable fluorescent active caspase-3/7 probe, FAM-DEVD-FMK (FAM-FLICA Caspase-3/7 assay kit, ImmunoChemistry Technologies, Davis, CA, USA), which detects early stage of apoptosis, was added to PEP51-expressed cells on 9.5mm-wells of multi-well glass-bottom dish for 30 min at 37°C. The stained cells were washed three times with 0.1M PBS pH 7.4, and fixed with 4% PFA in PBS for 30 min at room temperature. Subsequently, the cells were washed three times with TBS-T, blocked with 1% BSA for 30 min, and then treated with the anti-PEP51 antibody conjugated HiLyte Fluor 555 with an Ab-10 rapid fluorescein labeling kit (Dojindo) (diluted to 1:500) in TBS-T for 30 min. The signals of the active caspase-3/7 probe, FAM-DEVD-FMK and immunofluorescence staining were visualized using a confocal laser scanning microscope (Fluoview FV3000, Olympus, Tokyo, Japan) as described previously (15, 16).

### Detection of an apoptosis marker and PEP51 expression in *Ciona* follicles

According to previous *in vivo* uptake studies on *Ciona* follicles (6, 26, 27), follicle cells were enzymatically removed (0.2% actinase E in ASW) from the fractionated follicles for 10 min at room temperature by orbital shaking. After washing, the active caspase-3/7 probe, FAM-DEVD-FMK (FAM-FLICA caspase-3/7 assay kit, ImmunoChemistry Technologies) was added to the defolliculated follicles for 30 min at room temperature. The stained follicles were washed with 0.1M PBS pH 7.4, and fixed with 4% PFA in PBS at 4 °C for 16 hours. The fixed follicles were washed three times with TBS-T, blocked with 1% BSA for 30 min, and then treated with HiLyte Fluor 555-conjugated anti-PEP51 antibody (diluted to 1:200) in TBS-T for 30 min at room temperature. The signals of the active caspase-3/7 probe and immunofluorescence staining were visualized using a confocal laser scanning microscope (Fluoview FV3000, Olympus), as described above. For flow cytometry of test cells, the stained early stage III follicles were enzymatically disaggregated (0.1% trypsin, 0.5% collagenase, and 0.2% actinase E in ASW) for 15 min at room temperature by orbital shaking, and cells were passed through a 20-μm cell strainer (Sysmex) after adding 5% BSA. The stained test cells were fixed with 4% PFA in 0.1M PBS pH 7.4 at room temperature for 30 min. The fixed cells were washed three times with TBS-T, blocked with 1% BSA for 30 min, and then treated with HiLyte Fluor 555-conjugated anti-PEP51 antibody (diluted to 1:500) in TBS-T for 30 min at room temperature. After washing with TBS-T, the stained test cells were analyzed using BD FACSMelody (BD Biosciences).

### Statistical analysis

Results are expressed as means ± standard error for the indicated number of observations. Data were analyzed by one-way analysis of variance with Dunnett’s error protection. Differences were accepted as significant for *P <0.05*.

## Results

### Identification and localization of PEP51 in *Ciona* follicles

**Figure 1:**
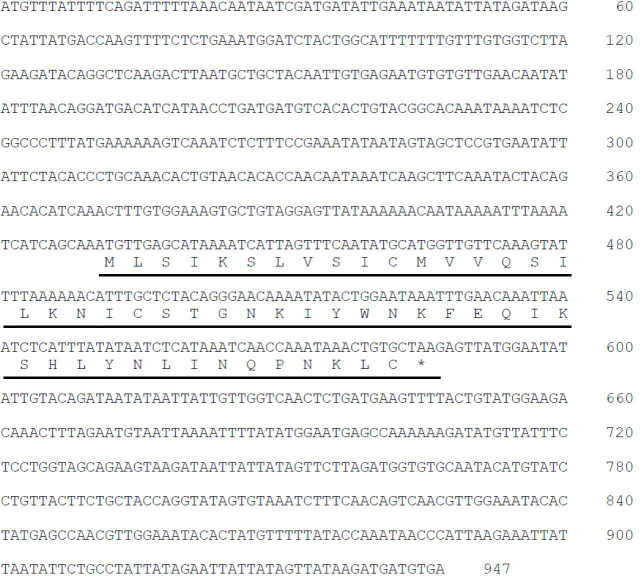
Detection of *Pep51* gene in *Ciona intestinalis* Type A.. Nucleotide sequence and deduced amino acid sequence of *Ciona* PEP51 cDNA. The sequence of PEP51 is indicated by underlined.

**Figure 2:**
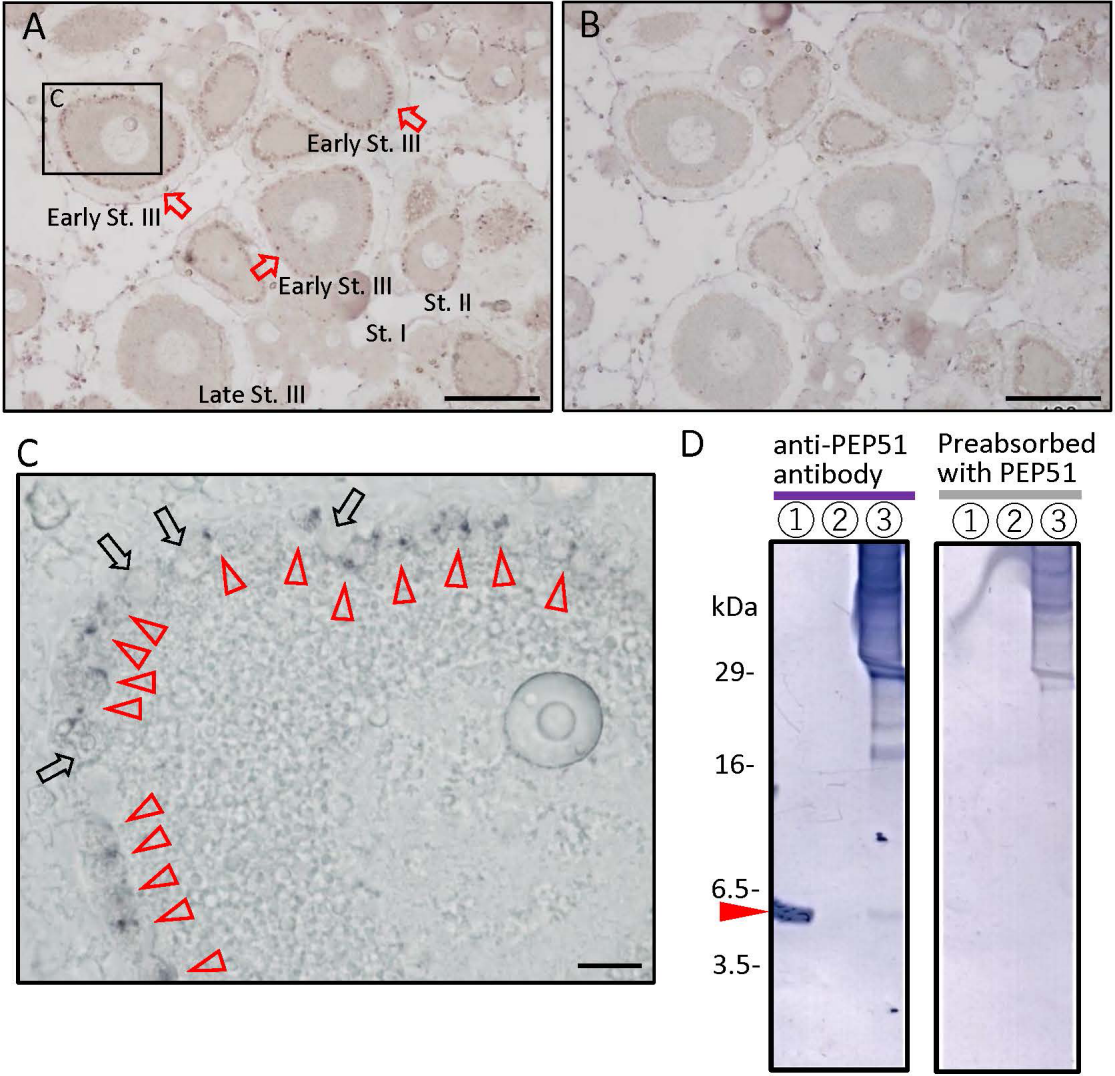
Localization of PEP51 in the *Ciona* ovary. **(A)** The anti-PEP51 antibody bound to fixed 7-μm sections of the ovaries detected the presence of PEP51 in early stage III (early-St. III) follicles. *Red arrows* indicate PEP51-immunoreactive follicles. Positive cells were detected in three separate experiments. **(B)** No specific immunostaining was observed using the preabsorbed anti-PEP51 antibody. *Scale bars*, 100 μm. **(C)** Magnified image of boxed area in A. *Red arrow heads* indicate anti-PEP51 immunoreactive test cells. Arrows indicate PEP51-non-expressed test cells. *Scale bar*, 10 μm. **(D)** Proteins in *Ciona* follicles were prepared from stage III follicles, and then immunoblotted with the anti-PEP51 antibody. *Red arrow heads* indicate the specific band for 10 ng synthetic PEP51 peptide (lane 1), and proteins from stage III follicles (lane 3) were detected at 6 kDa. Lane 2 is blank. Non-specific immunostaining was observed using the preabsorbed anti-PEP51 antibody (right panel). Several bands around 18-30 kDa, which were not detect with the preabsorbed antibody, suggest formation of complexes with PEP51 in stage III follicles (left panel).

We initially searched for the previous transcriptomes of the *Ciona intestinalis* Type A (accession ID; SRR24652239) and identified a 51-amino acid (51 aa) peptide-encoding gene (Ensembl gene ID: ENSCING00000021415; chromosome 3: 699,794 -700,784). No orthologs of the 51 aa peptide were detected on any database, indicating that this gene, named *Pep51*, is a *Ciona*-specific gene. The full-length cDNA was cloned from *Ciona* ovaries, and the open reading frame was found to encode a 51 aa peptide which completely accorded with that of the aforementioned sequence (**Figure 1**). To detect the translated PEP51 from the mRNA, we conducted immunohistochemical analysis of *Ciona* ovary sections using an anti-PEP51 antibody. As shown in **Figure 2A**, PEP51 was detected in many test cells of early stage III (post-vitellogenic) follicles. No positive signals were observed when antigen-absorbed antibody was applied, confirming the specificity of the immunostaining analysis (**Figure 2B**). In addition, a few PEP51-negative (PEP51(-)) test cells were observed in PEP51-positive (PEP51(+)) test cells of early stage III follicles (**Figure 2C**).

To elucidate the molecular weight of the immunoreactive molecule in stage III follicles, proteins were extracted from follicles of the corresponding stage by fractionating with a series of stainless-steel sieves, followed by western blotting using the anti-PEP51 antibody. A specific band was detected at 6 kDa, which coincided with the size of synthetic PEP51 with the calculated molecular weight of 5897.1175. (**Figure 2D**, left panel). Additionally, no bands were detected using the synthetic PEP51-absorbed anti-PEP51 antibody (**Figure 2D**, right panel), excluding the possibility of non-specific immunoreaction by the antibody. Additionally, several bands around 18-30 kDa were not detected with the preabsorbed antibody, suggesting the formation of some complexes with PEP51 in stage III follicles (**Figure 2D**, left panel). Taken together, these results indicate that PEP51 is expressed in the follicles, and that the amino acid sequence comprises 51 amino acids.

**Figure 3:**
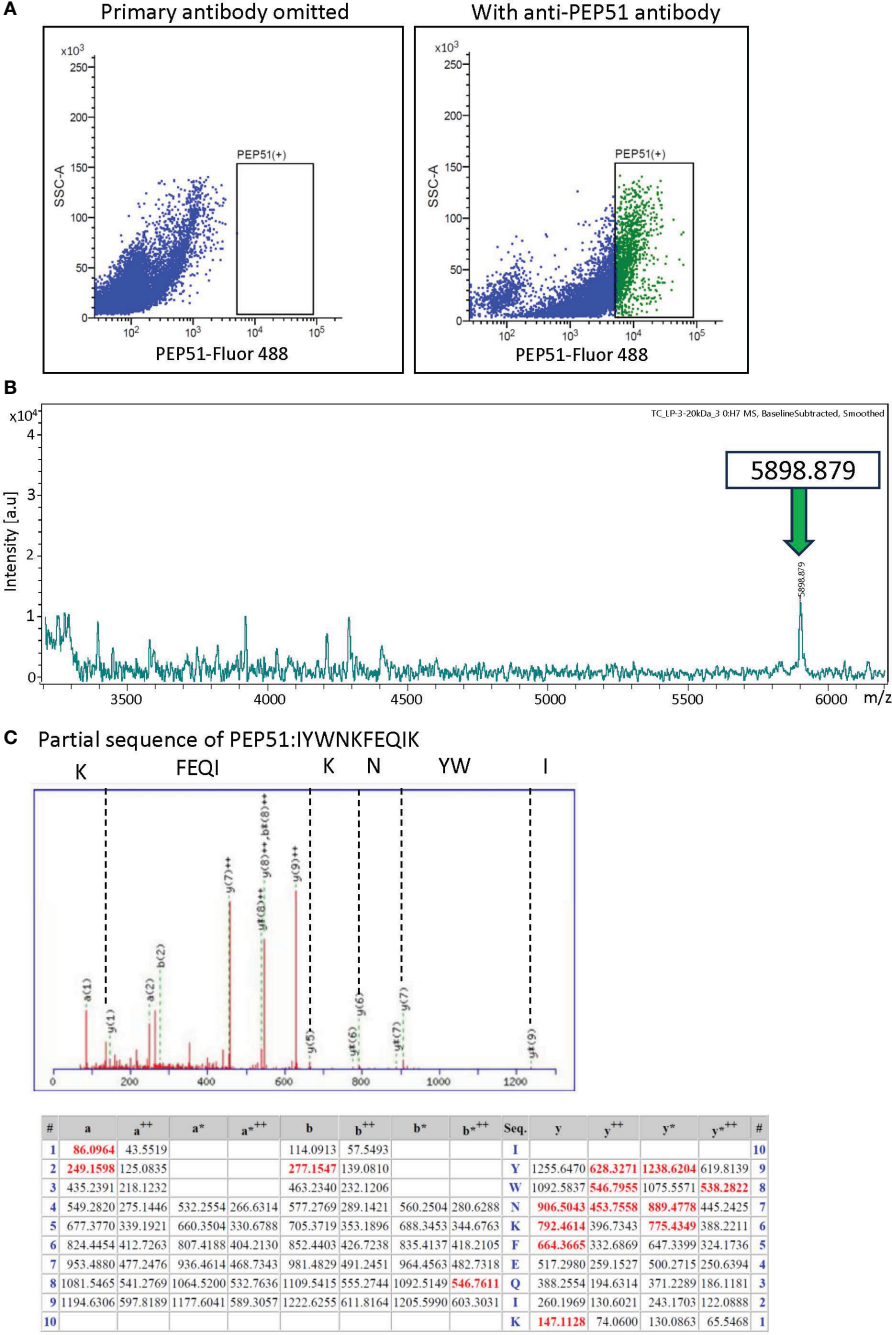
Identification of PEP51 from stage III follicles. **(A)** Expression of PEP51 was determined by flow cytometry. Cells stained for PEP51 were sorted by FACS for MALDI-TOF MS analysis. The results of flow cytometry are based on three independent experiments. PEP51-Alexa Fluor 488 signals were plotted against SSC-A signals. **(B)** Extracted endogenous peptides in the sorted cells were measured using MALDI-TOF MS. MALDI-TOF MS analysis of the extract allowed the detection of a peak corresponding to an MS value of 5898.879 [M + H]^+^ for PEP51. **(C)** Partial sequence of PEP51 obtained by tryptic digestion of the band corresponding with 6kDa on tricine-SDS-PAGE of the stage III follicle lysate was IYWNKFEQIK. MS/MS peaks were detected using the ESI Q-TOF MS instrument. y-type ion and several characteristic fragment ions are labeled. The observed peaks of the PEP51 fragment are shown in red. The symbol ++ indicates divalent ions. Fragments that lost ammonia (-17 Da) are denoted as a*, b* and y*.

Furthermore, FACS with the anti-PEP51 antibody and Alexa Fluor 488-conjugated secondary antibody was used to isolate PEP51(+) cells from stage-III follicles. We collected 1,150,000 cells in the PEP51(+) area shown in **Figure 3A**. Subsequently, endogenous peptides extracted from the sorted PEP51(+) cells were analyzed using matrix-assisted laser desorption/ionization time of flight mass spectrometry (MALDI-TOF MS), leading to the detection of a peak corresponding to an MS value of 5898.879 [M + H]^+^ for PEP51 (**Figure 3B**). Moreover, nanoLC Q-TOF MS/MS detected the IYWNKFEQIK sequence in tryptic digests of the peptide corresponding with a size of 6kDa, which completely corresponded with the partial sequence of PEP51 (**Figure 3C**). These analyses confirmed the presence of 51 aa full-length sequence of PEP51 in the test cells of stage III follicles. Moreover, intact endogenous PEP51 was electrophorized in accordance with synthetic PEP51 (**Figure 2D**), and the MS value of endogenous PEP51 (5898.879) (**Figure 3**) coincides with the calculated molecular weight of 5897.1175, indicating that PEP51 in test cells undergoes no post-translational modifications such as signal peptide cleavage or glycosylation. Collectively, we conclude that PEP51 is a *Ciona* ovarian intracellular factor in test cells but not peptide hormones or secretory proteins with post-translational modification.

### Subcellular localization of PEP51 in *Ciona* follicles

**Figure 4:**
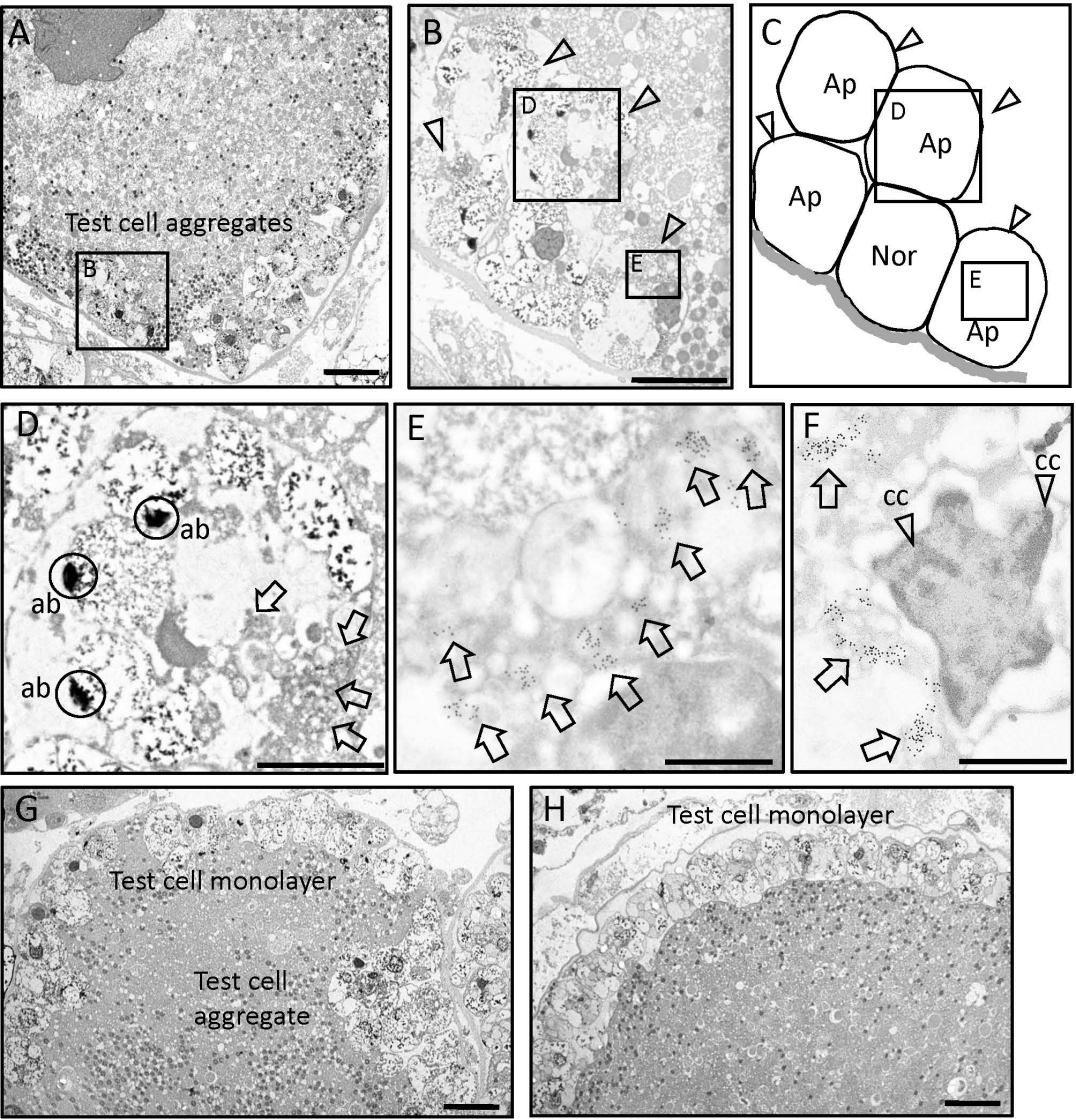
Immunoelectron microscopy on ultra-thin sections of stage III follicles. **(A)** Transmission electron micrographs of early stage III follicle with the aggregates of test cells. *Scale bar*: 10 μm. **(B)** A test cell aggregate outlined in panel A is shown at higher magnification. *Arrow heads* indicate anti-PEP51 immunoreactive test cells embedded in the oocyte at early stage III follicles. *Scale bar*: 10 μm **(C)** Illustration of apoptotic test cell (Ap) and normal test cell (Nor) in **(B)**. **(D)** Magnified image of boxed area in **(B)** *Arrows* indicate anti-PEP51 immunoreactivities in test cell, which exhibited apoptotic body-like constituents (ab). *Circles* indicate abs. *Scale bar*: 3 μm. **(E)** The region outlined in **(B)** is shown at higher magnification. A*rrows* indicate anti-PEP51-immunogold particles localized on small granules in the cytosol of test cell. *Scale bar*: 1 μm **(F)** PEP51(+) cells also show another feature of apoptosis, chromatin condensation (cc). A*rrows* indicate anti-PEP51-immunogold particles. A*rrow heads* indicate ccs. *Scale bar*: 1 μm. **(G)** Transmission electron micrographs of follicles between early stage III and late-stage III, which harbor test cell aggregates and a monolayer. *Scale bar*: 10 μm. **(H)** Transmission electron micrographs of a late stage III follicle. PEP51(-) test cells form a monolayer surrounding oocytes. *Scale bar*: 10 μm.

Subsequently, the subcellular localization of PEP51 was analyzed by immunoelectron microscopy using the anti-PEP51 antibody on the *Ciona* ovaries. Transmission electron micrographs show the characteristic localization of PEP51 in stage III follicles (**Figures 4A–G**). A greater number of anti-PEP51-immunogold particles were observed in test cells forming cellular aggregates embedded at early stage III follicles (**Figures 4A–G**). Higher magnification revealed localization of PEP51 in small cytosolic granules in the test cells (**Figures 4D–F**). Notably, numerous PEP51(+) test cells in early stage III follicles exhibited features typical of apoptosis including apoptotic body-like cellular constituents and chromatin condensation (**Figures 4B, D, F**), as seen in a previous study (26). Furthermore, PEP51(+) test cells were found to surround PEP51(-) (normal) test cells in the cellular aggregates in early stage III (**Figures 4A–C, G**), and such test cell aggregates were not observed in late stage III (**Figure 4H**), supporting the view that PEP51(+) test cells forming the aggregates in early stage III either disappeared or were eliminated at late stage III. Altogether, the localization of anti-PEP51-immunogold particles suggested some roles of PEP51 in the regulation of apoptosis in test cells of early stage III follicles.

### Activation of caspase-3/7 in PEP51-expressing test cells in developing *Ciona* follicles

**Figure 5:**
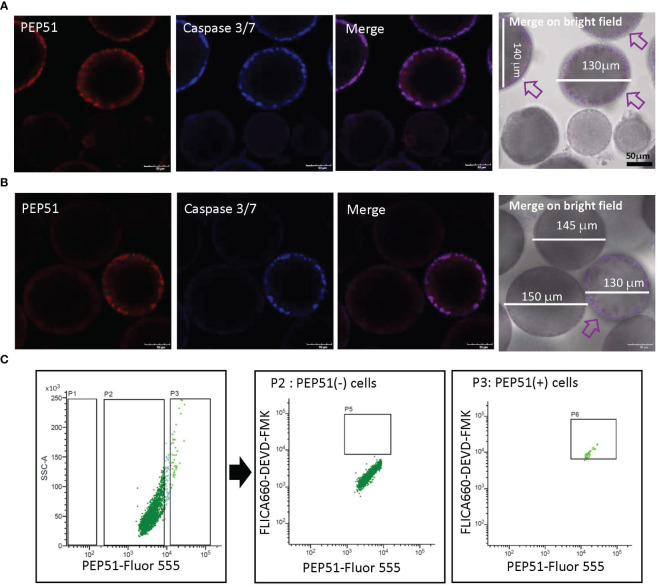
PEP51 expression and apoptosis detection in test cells of *Ciona* follicles. **(A)** Confocal microscope observation verified that PEP51 are colocalized with an apoptosis marker, FLICA660-DEVD-FMK exclusively in test cells of early stage III follicles (defolliculated follicles of around 130 μm in diameter). Red: HiLyte Fluor 555-conjugated anti-PEP51 antibody, Blue: FLICA660-DEVD-FMK (active caspase-3/7 probe). Arrows indicate PEP51-expressing follicles. Test cells of stage II follicles (defolliculated follicles of around 110 μm in diameter) did not show signal. **(B)** Test cells of late stage III (defolliculated follicles of around 150 μm in diameter) to stage IV follicles (defolliculated follicles of around 170 μm in diameter) did not show signal. An arrow indicates PEP51-expressing follicle. *Scale bars*: 50 μm. These results were obtained by five independent experiments. **(C)** Flow cytometry of test cells dissociated from early stage III follicles stained with the Fluor 555-conjugated anti-PEP51 antibody and the probe of active caspase-3/7 (FLICA660-DEVD-FMK). PEP51-Fluor 555 signal plotted against the FLICA660-DEVD-FMK signal. Areas P2 and P3 indicate PEP51(-) cells and PEP51(+) cells, respectively. Few FLICA660-DEVD-FMK signal-positive cells (P5, center panel) were detected in PEP51(-) cells. In contrast, most PEP51(+) cells show high FLICA660-DEVD-FMK signal (area P6, right panel). Collectively, quantification of the outputs by the FACS analyses revealed that 97.62% of PEP51(+) cells and 0.81% of PEP51(-) cells in early stage III follicles showed activation of caspase-3/7, respectively. These results were obtained by three independent FACS analyses.

Previous studies demonstrated apoptosis of test cells residing within mature *Ciona* eggs (stage IV follicles) in the oviduct by detection of canonical apoptotic markers, active pan-caspases (26, 27). However, apoptosis of test cells in developing *Ciona* follicles in the ovary has yet to be investigated. We thus examined the expression of PEP51 and apoptosis of test cells in follicles using the HiLyte Fluor 555-conjugated anti-PEP51 antibody, and a cell-permeable far-red fluorescent 660 dye-labeled probe for the active caspase-3/7, FLICA660-DEVD-FMK. Confocal laser scanning microscopy verified that PEP51 was colocalized with the apoptosis marker in test cells of early stage III follicles (defolliculated follicles of around 130 μm in diameter) (**Figure 5A**). In addition, such an apoptotic signal was not detected in test cells of stage II follicles (defolliculated follicles of around 110 μm in diameter) nor in late stage III (defolliculated follicles of around 150 μm in diameter) to stage IV follicles (defolliculated follicles of around 170 μm in diameter) (**Figure 5B**). These results demonstrated that caspase activation occurred in most PEP51(+) test cells of early stage III follicles (**Table 1**). To quantitatively analyze this confocal microscope observation, we performed flow cytometry for test cells dissociated from the stained early stage III follicles. FACS analysis revealed that caspase activation occurred in 97.62% of PEP51(+) test cells and 0.81% of PEP51(-) test cells in early stage III follicles, respectively (**Figure 5C**). Taken together, these results suggested the functional role for PEP51 in the induction of apoptosis via activation of caspase-3/7.

**Table 1:** The ratio of double signal-positive *Ciona* follicles at each stage.

Diameter of defolliculated follicles (μm)	Double positive signals (%)
Stage I: less than 90: Stage II: around 110 Early stage III: around 130 Late stage III: around 150 Stage IV: around 170	0 5.27 ± 1.50 100 7.63 ± 2.11 0

Data are means ± SE of three independent experiments using 30 defolliculated follicles.

### Induction of apoptosis by PEP51 in multiple cell lines

**Figure 6:**
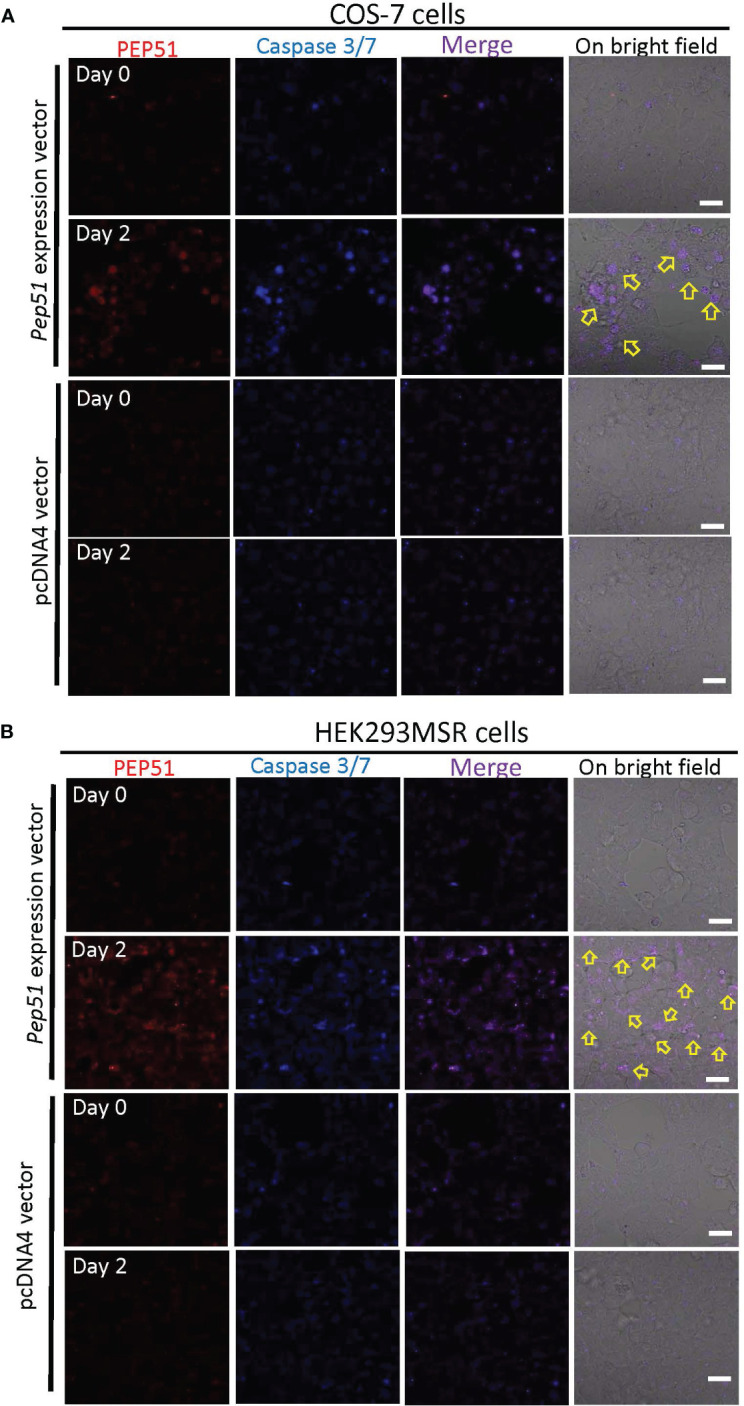
Activation of caspase-3/7 in PEP51-expressing cells. **(A)** After the transfection with PEP51-expression vector into COS-7 cells for 2 days, confocal laser scanning microscopy revealed the colocalization of PEP51 and the apoptosis marker, FLICA660-DEVD-FMK (active caspase-3/7 probe) signals. Red: HiLyte Fluor 555-conjugated anti-PEP51 antibody, Blue: FLICA660-DEVD-FMK. Arrows indicate apoptosis in PEP51-transfected cells, whereas no positive signal was observed in the COS-7 cells transfected with an empty pcDNA4 vector. **(B)** After the transfection with PEP51-expression vector into HEK293MSR cells for 2 days, colocalization of PEP51 and FLICA660-DEVD-FMK signals were detected, whereas no positive signal was observed in HEK293MSR cells transfected with an empty pcDNA4 vector. *Scale bars*: 20 μm. Each of the expression vector and an empty pcDNA4 vector (0.1 μg) was transiently transfected into 5×10^4^ cells in 9.5-mm wells of multi-well glass-bottom dish with Lipofectamine 2000.

To examine whether PEP51 directly induce caspase activation, we evaluated the effect of the PEP51 gene by transfection of *Pep51* expression vector into COS-7 cells and HEK293MSR cells and addition of the active caspase-3/7 probe, FLICA660-DEVD-FMK. The apoptosis marker was detected specifically in PEP51-transfected COS-7 cells (**Figure 6A**) at 2 days after transfection. Similar data were obtained using HEK293MSR cells (**Figure 6B**). In contrast, no positive signal was detected in the cells transfected with an empty pcDNA4 vector (**Figures 6A, B**). The ratio of caspase-3/7 signal-positive cells at 2 days after transfection is summarized in **Table 2**. These results provided evidence that PEP51 activated caspase-3/7.

**Table 2:** The ratio of caspase-3/7 signal-positive cells at 2 days after transfection.

COS-7 cells	Positive cells (%)
*Pep51* expression vector pcDNA4 empty vector Transfection medium alone	58.64 ± 5.25* 4.50 ± 1.28 3.61 ± 2.02

HEK293MSR cells	Positive cells (%)
*Pep51* expression vector pcDNA4 empty vector Transfection medium alone	63.59 ± 4.08* 3.16 ± 2.62 3.25 ± 1.95

Data are means ± SE of five independent experiments. Significance of differences between transfection medium alone group and vector transfected groups in each cell line was evaluated by one-way ANOVA with Dunnett error protection (*, *P* < 0.01).

## Discussion

In the present study, we identified a *Ciona*-specific peptide, PEP51, which is expressed in the cytosol of test cells in early stage III follicles, as an activator of caspase-3/7. PEP51 was expressed mainly in the test cells of early stage III follicles, which lack secretory granules (23, 25). These results suggest that PEP51 acts as an intracellular factor within test cells. Previous studies (28, 29) identified an ortholog of caspase-3, -6, -7 from *Ciona*, which conserved catalytic site (QACRG pentapeptide) of vertebrate caspase-3, -6, and -7. Additionally, these studies revealed an active form of *Ciona* caspase-3-like protein and caspase-3 enzymatic activity in a tissue of metamorphosis stage, and detected signals for activated caspases of apoptotic cells in the stage using fluorescent marker for active caspases (FITC-VAD-FMK and Red-DEVE-FMK) (28, 29). Collectively, these findings confirm that a caspase-3-like protein is activated during apoptosis in *Ciona* and conserves the substrate specificity.

**Figure 7:**
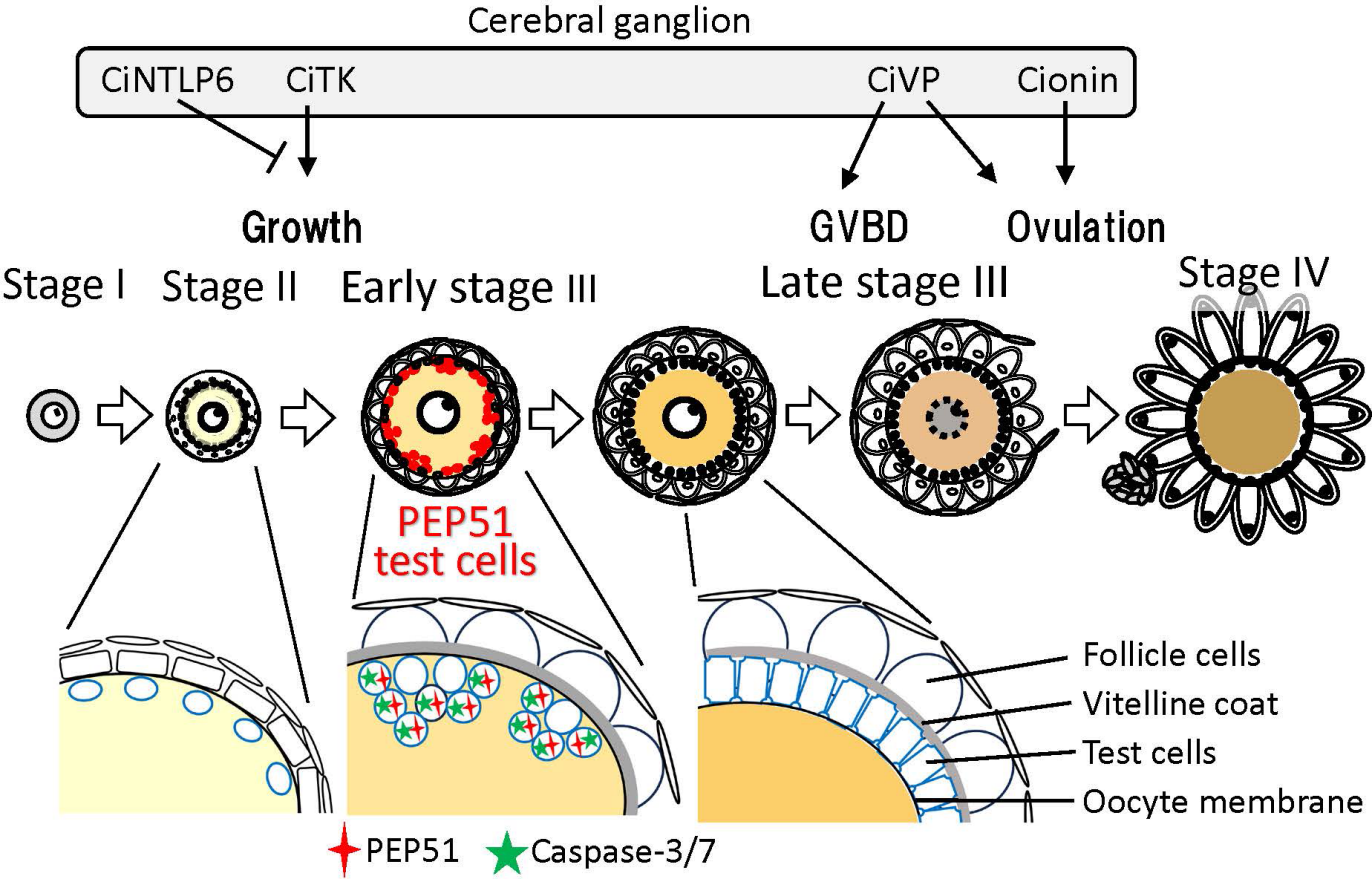
Schematic drawing of PEP51 expression and neuropeptidergic regulation of *Ciona* ovarian follicular growth, oocyte maturation, and ovulation. In stage II follicles, *Ciona* tachykinin (CiTK) promotes follicle growth from stage II to stage III. CiNTLP6 suppresses CiTK-induced gene expression and subsequent follicle growth. In late stage III follicles, *Ciona* vasopressin (CiVP) promotes oocyte maturation via GVBD and ovulation. Cionin also induces ovulation in stage III follicles. PEP51 is expressed in the vitellin coat of stage II follicles and test cells embedded in the oocyte membrane of early stage III follicles. Enlarged illustrations indicate the alteration of test cell organization and PEP51 expression profiles between stage II and late stage III. PEP51(-) test cells disperse in the oocyte membrane of stage II. A few PEP51(-) test cells reside in the aggregates of PEP51(+) test cells, which activate caspase-3/7 in early stage III follicles. PEP51(-) test cells form a monolayer covering oocytes in the perivitelline space of late stage III follicles.

To date, different distributions and functions of test cells during follicle development in ascidians has been reported. Test cells in the perivitelline space of stage IV follicles (mature eggs) move and attach to the developing embryos after fertilization, and persist on the hatched tadpole larvae in *Halocynthia roretzi* and *C. intestinalis* Type A (24–26, 30, 31). Moreover, test cells disperse in the surface of oocytes at stage II, aggregate at early stage III, and form a monolayer surrounding oocytes at late stage III (4, 5, 23, 24). Notably, our present study revealed that most test cells in early stage III follicles express PEP51 and undergo apoptosis, whereas PEP51 was not observed in test cells at late stage III (**Figures 2A**, **4H**, and **5B**). These findings suggest that most of the test cells were replaced from early stage III to late stage III of the *Ciona* follicles (**Figure 4G**), and exerted different functions at the respective stages. The specific expression of the CiTK receptor gene in test cells of stage II follicles (8, 9), and *Ciona* GnRH receptors, CiGnRHR-1, -2, and -4, in test cells of late stage III follicles (15, 16, 32, 33), and the morphological changes such as the disappearance of secretory granules and the development of large vacuoles in test cells between stage II and early stage III (23, 24), support the view that test cells possess different functions at each follicle growth and maturation stage in *Ciona*. Altogether, the present study suggests that PEP51 participates in the replacement of test cells via induction of apoptosis, leading to a *Ciona*-specific alteration of the biological roles of test cells during follicle development (**Figure 7**).

Recent studies on various animals have demonstrated that apoptotic cells stimulate the proliferation of neighboring cells, termed apoptosis-induced proliferation or compensatory proliferation (34, 35). Furthermore, combined with the involvement of caspase-driven apoptosis in tissue regenerations of vertebrates and invertebrates (35, 36), the present data imply that PEP51-induced apoptosis via activation of caspases stimulates the replacement of test cells from early stage III to late stage III via compensatory proliferation. Caspases-dependent apoptosis consists of complexed proteolytic signaling cascades by multiple endogenous factors including caspase initiators, caspase-2, -8, -9, -10, -11, and -12 (37). Furthermore, multiple pro-apoptotic molecules and anti-apoptotic molecules, positively and negatively regulate the initiator and effector caspases (37). Elucidation of the molecular mechanism underlying the activation of caspase-3/7 by PEP51 is underway. Moreover, a few PEP51(-) test cells reside in the aggregates of PEP51(+) apoptotic test cells of early stage III follicles (**Figures 2A, C**, and **4A–C**), and only PEP51(-) test cells form a monolayer surrounding oocytes in late stage III follicles (**Figures 2A** and **4H**). These PEP51 expression profiles and the alteration of test cell organization support the notion that induction of caspase-3/7-directed apoptosis by PEP51 is functionally associated with the compensatory proliferation by which apoptotic PEP51(+) test cells stimulate the proliferation of neighboring PEP51(-) test cells. Moreover, the expression of PEP51 in early stage III follicles (**Figures 2A, C**, **4A–C**, and **5A, B**), which precedes oocyte maturation via germinal vesicle breakdown (GVBD) in late stage III, suggests some biological roles of PEP51(+) test cells-induced apoptosis during follicle maturation. Molecular and physiological analyses of the functions of PEP51 in the compensatory proliferation in *Ciona*, including PEP51 genome editing, are currently in progress.

No orthologs of PEP51 were detected on any database, indicating that the PEP51 gene is *Ciona*-specific. Therefore, the present study sheds new light on the possibility that species-specific molecules participate in regulation of follicle development in chordates. Also of interest is whether the regulation of follicular development via an intracellular ovarian peptide-directed apoptotic process of accessory cells is *Ciona*-specific or evolutionarily conserved throughout Chordata or the animal kingdom, although no ovarian peptides have been shown to induce apoptosis in an animal.

In conclusion, the present study identified a novel species-specific ovarian peptide, PEP51, which induces the activation of caspase-3/7, and is likely to be involved in disappearance of the aggregates of PEP51(+) test cells in post-vitellogenic follicles. Combined with the phylogenetic position of *Ciona* as the closest sister group of vertebrates, these data suggest that such an ovarian species-specific peptide plays key roles in the regulation and evolution of follicle development in chordates.

## Data availability statement

The datasets presented in this study can be found in online repositories. The names of the repository/repositories and accession number(s) can be found below: https://www.ncbi.nlm.nih.gov/sra/, (accession ID: SRR24652239).

## Ethics statement

Ethical approval was not required for the studies on animals in accordance with the local legislation and institutional requirements.

## Author contributions

TS: Conceptualization, Investigation, Visualization, Writing – original draft, Writing – review & editing. TY: Data curation, Methodology, Investigation, Writing – review & editing. TW: Data curation, Methodology, Writing – review & editing. AH: Investigation, Writing – review & editing. AS: Investigation, Methodology, Writing – review & editing. TO: Data curation, Investigation, Methodology, Supervision, Writing – review & editing. SM: Data curation, Investigation, Methodology, Supervision, Writing – review & editing. TK: Investigation, Methodology, Supervision, Writing – review & editing. YS: Investigation, Methodology, Supervision, Writing – review & editing. TT: Investigation, Methodology, Supervision, Writing – review & editing. HS: Conceptualization, Data curation, Funding acquisition, Project administration, Supervision, Writing – original draft, Writing – review & editing, Investigation, Methodology.

## References

[1] Edson MA Nagaraja AK Matzuk MM. The MamMalian ovary from genesis to revelation. Endocr Rev (2009) 30:624–712. doi: 10.1210/er.2009-0012 19776209 PMC2761115

[2] Von Stetina JR Orr-Weaver TL. Developmental control of oocyte maturation and egg activation in metazoan models. Cold Spring Harb Perspect Biol (2011) 3:a005553. doi: 10.1101/cshperspect.a005553 21709181 PMC3179337

[3] Satoh N Rokhsar D Nishikawa T. Chordate evolution and the three-phylum system. Proc Biol Sci. B (2014) 281:20141729. doi: 10.1098/rspb.2014.1729 PMC4211455 25232138

[4] Lambert C. Ascidian follicle cells: Multifunctional adjuncts to maturation and development. Dev Growth Differ (2009) 51:677–86. doi: 10.1111/j.1440-169X.2009.01127 19703210

[5] Prodon F Chenevert J Sardet C. Establishment of animal-vegetal polarity during maturation in ascidian oocytes. Dev Biol (2006) 290:297–311. doi: 10.1016/j.ydbio.2005.11.025 16405883

[6] Matsubara M Shiraishi A Osugi T Kawada T Satake H. The Regulation of oocyte maturation and ovulation in the closest sister group of vertebrates. Elife (2019) 8::e49062. doi: 10.7554/eLife.49062 31573508 PMC6786877

[7] Matsubara S Shiraishi A Osugi T Kawada T Satake H. Fractionation of ovarian follicles and in *vitro* oocyte maturation and ovulation assay of *Ciona intestinalis* Type A. Bio Protoc (2020) 10:e3577. doi: 10.21769/BioProtoc.3577 PMC7842689 33659547

[8] Aoyama M Kawada T Fujie M Hotta K Sakai T Sekiguchi T. A novel biological role of tachykinins as an up-regulator of oocyte growth: identification of an evolutionary origin of tachykininergic functions in the ovary of the ascidian, *Ciona intestinalis* . Endocrinology (2008) 149:4346–56. doi: 10.1210/en.2008-0323 18483149

[9] Aoyama M Kawada T Satake H. Localization and enzymatic activity profiles of the proteases responsible for tachykinin-directed oocyte growth in the protochordate. Ciona intestinalis. Peptides (2012) 34:186–92. doi: 10.1016/j.peptides.2011.07.019 21827805

[10] Kawada T Ogasawara M Sekiguchi T Aoyama M Hotta K Oka K. Peptidomic analysis of the central nervous system of the protochordate, *Ciona intestinalis*: homologs and prototypes of vertebrate peptides and novel peptides. Endocrinology (2011) 152:2416–27. doi: 10.1210/en.2010-1348 21467196

[11] Osugi T Miyasaka N Shiraishi A Matsubara S Satake H. Cionin, a vertebrate cholecystokinin/gastrin homolog, induces ovulation in the ascidian *Ciona intestinalis* Type A. Sci Rep (2021) 11:10911. doi: 10.1038/s41598-021-90295-3 34035343 PMC8149874

[12] Kawada T Shiraishi A Matsubara S Hozumi A Horie T Sasakura Y and Satake H. Vasopressin promoter transgenic and vasopressin gene-edited ascidian, *Ciona intestinalis* Type A (*Ciona robusta*): innervation, gene expression profiles, and phenotypes. Front Endocrinol (2021) 12:668564. doi: 10.3389/fendo.2021.668564 PMC8135067 34025581

[13] Kawada T Osugi T Matsubara S Sakai T Shiraishi A Yamamoto T and Satake H. Omics studies for the identification of ascidian peptides, cognate receptors, and their relevant roles in ovarian follicular development. Front Endocrinol (2022) 13:858885. doi: 10.3389/fendo.2022.858885 PMC8936170 35321341

[14] Satake H. Kobayashi Award 2021: Neuropeptides, receptors, and follicle development in the ascidian, *Ciona intestinalis* Type A: New clues to the evolution of chordate neuropeptidergic systems from biological niches. Gen Comp Endocrinol (2023) 337:114262. doi: 10.1016/j.ygcen.2023.114262 36925021

[15] Sakai T Aoyama M Kusakabe T Tsuda M Satake H. Functional diversity of signaling pathways through G protein-coupled receptor heterodimerization with a species-specific orphan receptor subtype. Mol Biol Evol (2010) 27:1097–106. doi: 10.1093/molbev/msp319 20026483

[16] Sakai T Aoyama M Kawada T Kusakabe T Tsuda M Satake H. Evidence for differential regulation of GnRH signaling via heterodimerization among GnRH receptor paralogs in the protochordate, *Ciona intestinalis* . Endocrinology (2012) 153:1841–9. doi: 10.1210/en.2011-1668 22294747

[17] Shiraishi A Okuda T Miyasaka N Osugi T Okuno Y Inoue J. Repertoires of G protein-coupled receptors for *Ciona*-specific neuropeptides. Proc Natl Acad Sci USA (2019) 116:7847–56. doi: 10.1073/pnas.1816640116 PMC6475428 30936317

[18] Robker RL Hennebold JD Russell DL. Coordination of ovulation and oocyte maturation: a good egg at the right time. Endocrinology (2018) 159:3209–18. doi: 10.1210/en.2018-00485 PMC6456964 30010832

[19] Duffy DM Ko C Jo M Brannstrom M Curry TE. Ovulation: parallels with inflammatory processes. Endocr Rev (2019) 40:369–416. doi: 10.1210/er.2018-00075 30496379 PMC6405411

[20] Orisaka M Miyazaki Y Shirafuji A Tamamura C Tsuyoshi H Tsang BK. The role of pituitary gonadotropins and intraovarian regulators in follicle development: A mini-review. Reprod Med Biol (2021) 20:169–75. doi: 10.1002/rmb2.12371 PMC8022101 33850449

[21] Takahashi T Hagiwara A Ogiwara K. Follicle rupture during ovulation with an emphasis on recent progress in fish models. Reproduction (2019) 157:R1–R13. doi: 10.1530/REP-18-0251 30394703

[22] Takahashi T Ogiwara K. cAMP signaling in ovarian physiology in teleosts: a review. Cell Signal (2023) 101:110499. doi: 10.1016/j.cellsig.2022.110499 36273754

[23] Mancuso V. An electron microscope study of the test cells and follicle cells of *Ciona intestinalis* during oogenesis. Acta Embryol. Morphol. Exp (1965) 8:239–66. 5899521

[24] Takamura K Ueda Y Irie U Yamaguchi Y. Immunohistology with antibodies specific to test cells in the ascidian *Ciona intestinalis* suggests their role in larval tunic formation. Zoological Sci (1996) 13:241–51. doi: 10.2108/zsj.13.241

[25] Okada T Takamura K Yamaguchi Y Yamamoto M. Secretory function of the test cell in larval tunic formation in the ascidian *Ciona intestinalis*: An immunoelectron microscopic study. Zoological Sci (1996) 13:253–61. doi: 10.2108/zsj.13.253

[26] Maury B Martinand-Mari C Chambon JP Soulé J Degols G Sahuquet A. Fertilization regulates apoptosis of *Ciona intestinalis* extra-embryonic cells through thyroxine (T4)-dependent NF-kappaB pathway activation during early embryonic development. Dev Biol (2006) 289:152–65. doi: 10.1016/j.ydbio.2005.10.021 16313896

[27] Martinand-Mari C Maury B Rousset F Sahuquet A Mennessier G Rochal S. Topological control of life and death in non-proliferative epithelia. PLoS One (2009) 4:e4202. doi: 10.1371/journal.pone.0004202 19145253 PMC2625397

[28] Chambon JP Soule J Pomies P Fort P Sahuquet A Alexandre D. Tail regression in *Ciona intestinalis* (Prochordate) involves a caspase-dependent apoptosis event associated with ERK activation. Development (2002) 129:3105–14. doi: 10.1242/dev.129.13.3105 12070086

[29] Comes S Locascio A Silvestre F d'Ischia M Russo GL Tosti E. Regulatory roles of nitric oxide during larval development and metamorphosis in. Ciona intestinalis. Dev Biol (2007) 306:772–84. doi: 10.1016/j.ydbio.2007.04.016 17499701

[30] Satoh N Numakunai T Hori R. Behavior and cellular morphology of the test cells during embryogenesis of the ascidian. Halocynthia roretzi. J Morphol. (1982) 171:219–23. doi: 10.1002/jmor.1051710209 30081612

[31] Totsuka NM Kuwana S Sawai S Oka K Sasakura Y Hotta K. Distribution changes of non-self-test cells and self-tunic cells surrounding the outer body during Ciona metamorphosis. Dev Dynamics (2023) 1–12. doi: 10.1002/dvdy.636 37341471

[32] Sakai T Shiraishi A Kawada T Matsubara S Aoyama M Satake H. Invertebrate gonadotropin-releasing hormone-related peptides and their receptors: an update. Front Endocrinol (Lausanne) (2017) 8:217. doi: 10.3389/fendo.2017.00217 28932208 PMC5592718

[33] Sakai T Yamamoto T Matsubara S Kawada T Satake H. Invertebrate gonadotropin-releasing hormone receptor signaling and its relevant biological actions. Int J Mol Sci (2020) 21:8544. doi: 10.3390/ijms21228544 33198405 PMC7697785

[34] Moreno-Celis U García-Gasca T Mejía C. Apoptosis-induced compensatory proliferation in cancer. In: Sergi CM, editor. Metastasis. Brisbane (AU: Exon Publications (2021). doi: 10.36255/exon-publications.metastasis.apoptosis-proliferation 35679456

[35] Fogarty CE Bergmannn A. Killers creating new life: caspases drive apoptosis induced proliferation in tissue repair and disease. Cell Death Differ (2017) 24:1390–400. doi: 10.1038/cdd.2017.47 PMC5520457 28362431

[36] Eskandari E Eaves CJ. Paradoxical roles of caspase-3 in regulating cell survival, proliferation, and tumorigenesis. J Cell Biol (2022) 221:e202201159. doi: 10.1083/jcb.202201159 35551578 PMC9106709

[37] Sahoo G Samal D Khandayataray P Murthy MK. A review on caspases: key regulators of biological activities and apoptosis. Mol Neurobiol (2023) 22. doi: 10.1007/s12035-023-03433-5 37349620

